# Rethinking the Approach to Preclinical Models of Anorexia Nervosa

**DOI:** 10.1007/s11920-022-01319-2

**Published:** 2022-02-11

**Authors:** Marie François, Lori M. Zeltser

**Affiliations:** 1 Division of Molecular Genetics, Naomi Berrie Diabetes Center, Columbia University Irving Medical Center, New York, USA; 2 Department of Pathology and Cell Biology, Columbia University Irving Medical Center, New York, USA

**Keywords:** Activity-based anorexia, Animal models, Anorexia nervosa, Cognitive inflexibility, Perseverative behavior, Neural circuits

## Abstract

**Purpose of Review:**

The goal of this review is to describe how emerging technological developments in pre-clinical animal research can be harnessed to accelerate research in anorexia nervosa (AN).

**Recent Findings:**

The activity-based anorexia (ABA) paradigm, the best characterized animal model of AN, combines restricted feeding, excessive exercise, and weight loss. A growing body of evidence supports the idea that pathophysiological weight loss in this model is due to cognitive inflexibility, a clinical feature of AN. Targeted manipulations that recapitulate brain changes reported in AN — hyperdopaminergia or hyperactivity of cortical inputs to the nucleus accumbens — exacerbate weight loss in the ABA paradigm, providing the first evidence of causality.

**Summary:**

The power of preclinical research lies in the ability to assess the consequences of targeted manipulations of neuronal circuits that have been implicated in clinical research. Additional paradigms are needed to capture other features of AN that are not seen in ABA.

## Introduction

A suite of newly developed tools in pre-clinical (animal) models can help overcome several obstacles to understanding brain mechanisms of anorexia nervosa (AN). Advances in current methods can characterize, map, and manipulate neural circuits with unprecedented cellular precision [1–3]. Application of these cutting-edge tools is leading to rapid advances in the understanding of many psychiatric disorders, such as schizophrenia and autism spectrum disorder [4]. To leverage these tools to study AN, relevant preclinical models are needed. Historically, animal models have attempted to recapitulate as many features of AN as possible. In this review, we use the best characterized preclinical model of AN, activity-based anorexia (ABA), to illustrate limitations in the traditional approach to animal models and to highlight emerging approaches that use the ABA model to identify circuits and molecular pathways responsible for discrete aspects of AN.

## ABA Paradigm: Classical Approaches

In the ABA paradigm, rodents are provided with continuous access to a running wheel and are then exposed to time-restricted feeding, such that access to food is confined to the first 1–2 h of the dark phase. Instead of eating during this limited feeding period, some rodents paradoxically choose to run; this leads to a precipitous decrease in body weight and decrease in survival over the next few days [5]. This relatively simple model captures features of AN, including increased susceptibility in adolescence, physiological and hormone changes associated with weight loss, excessive exercise, and caloric restriction (although it should be acknowledged that limited access to food is not the case in humans) [6]. Researchers have particularly touted the ABA as a powerful system to study the interaction between hyperactivity, caloric restriction and weight loss that is observed in AN [7–10].

Similar to clinical research, rodent studies compare ABA groups with unaffected “healthy comparison” groups. The comparison groups consist of ad libitum fed animals; a weight-recovered group is sometimes included as well. These studies demonstrate that, like in patients with AN, ABA rodents have decreased brain volume that is reversed by re-feeding [11–14, 15•]. Weight loss is also associated with increased markers of oxidative stress in the prefrontal cortex that is reversed by weight restoration [16]. The ABA model provided new insights into the impact of AN on the brain by identifying reductions in astrocyte number and proliferation in the cerebral cortex without a change in neuronal number [15•]. This finding challenges the existing assumption that deficits in the grey matter and white matter of patients with AN stemmed from decreased neurogenesis [17] and introduces a new potential mechanistic hypothesis involving a role for astrocytes in AN. Animal models, including ABA, have the potential to provide ways of distinguishing components of illness and differential brain effects. For example, by including control groups that may disentangle effects of exercise and caloric restriction on the brain.

A control group can be exposed to exercise alone, without weight loss, or to restricted feeding only, which produces the same amount of weight loss, but with less associated mortality [18, 19, 20•]. Comparisons between mice exposed to restricted feeding only vs. the ABA paradigm reveal that patterns of neuronal activation in the hypothalamus are largely driven by restricted feeding [21, 22]. While the inclusion of additional control groups is very powerful, it is also laborious.

Another strategy involves exposing a large cohort of animals through the ABA paradigm and then comparing the group that develops ABA (vulnerable) versus the group that does not (resilient). Vulnerable mice rapidly lose body weight within the first few days, while resilient mice adapt to an initial period of weight loss and maintain a stable weight [20•, 23]. Because the animals experience the same experimental conditions, it is easier to identify factors driving vulnerability. Some groups have shown that baseline running activity predicts susceptibility to ABA [24–26, 27••], but translating this observation to human behavior and physiology is not straightforward. Rodents adapt to a schedule of restricted feeding by increasing their locomotor activity immediately before access to food is expected [28]. This phenomenon, called “food anticipatory behavior,” reflects an evolutionarily advantageous adaptation to increase motivation to seek food when it is available in limited supplies [29]. Thus, food anticipatory behavior promotes caloric intake under restricted access paradigms and is associated with resilience to the ABA [20•]. On the other hand, some have posited that excessive running in the ABA model reflects a pathophysiological expansion of food anticipatory behavior [30]. Greater resolution in activity measurements is needed to resolve this issue.

Changes in synaptic transmission in the hippocampus are associated with vulnerability to ABA [31]. For example, there is a strong negative correlation between hippocampal expression of the glutamate transporter GLT1 and running wheel activity and weight loss in the ABA model [32•]. Loss of GLT1 function is sufficient to enhance wheel running activity and repetitive behavior [33••], consistent with the idea that the resulting increase in glutamate transmission promotes ABA vulnerability. At the same time, the two primary manipulations in the ABA model, voluntary exercise and caloric restriction, are associated with increases in hippocampal GLT1 expression and glutamate uptake that are predicted to decrease glutamate transmission [34, 35]. Thus, vulnerability to ABA could reflect low baseline levels of GLT1 and/or the failure to appropriately upregulate its expression in response to caloric restriction or activity. These animal models can identify behaviors, physiological adaptations, or biomarkers of susceptibility.

## ABA Paradigm: Moving Beyond Correlation to Causation

New powerful techniques can be used to physically target a specific brain region, and genetic tools can target a specific neuronal population within that region. These techniques can be used to replicate neuronal changes that have been observed in neuroimaging studies in patients with AN. For example, increased binding to the D2/D3 dopamine receptor (D2R/D3R) is observed in the ventral striatum of women who recovered from AN [36]. In animals, this finding is mimicked by overexpression of the D2R autoreceptor exclusively in the nucleus accumbens. This targeted manipulation produces localized hyperdopaminergia [37, 38] and accelerates weight loss in the ABA paradigm in females, but not in males [39••]. These findings are consistent with reports that hyperdopaminergia produced by genetic reductions in dopamine transporter expression increases susceptibility to the ABA paradigm in female mice [20•]. Understanding how D2R circuits influence metabolic adaptation to restricted feeding could provide novel insights into the mechanism underlying sex differences in susceptibility to AN [40, 41]. Moreover, further characterization of this overexpression model using other behavioral and physiological assays can help to identify additional features of AN that might be caused by increased D2R in the ventral striatum.

Neural circuits can also be manipulated with emerging neuroscience techniques. Pathway specific “chemogenetic” tools have yielded a particularly compelling experimental story relevant for AN [27••]. Chemogenetic tools are used to modulate activity of projections from the prefrontal cortex to the nucleus accumbens. This circuit was identified in human neuroimaging studies to show abnormal connectivity in acute AN and after weight restoration [42]. Stimulation of this circuit increases perseverative wheel running behavior and exacerbates weight loss [27••]. Conversely, inhibition of this pathway improves flexibility during early reversal learning by reducing perseverative responding and promotes food intake [27••]. This is consistent with the literature showing that this circuit modulates set shifting in rodents [43, 44]. Cognitive inflexibility is also seen in patients with AN [45–47] and in rodents in the ABA paradigm [48].

These observations raise the possibility that the ABA model captures a failure to adapt to restricted access to food in the face of preservative running behavior. In support of this idea, acclimation to restricted feeding before providing access to the running wheel suppresses hyperactivity in the ABA paradigm [19]. Additionally, the excessive running and weight loss phenotypes of the ABA model are no longer observed when the total time with access to food is distributed across several shorter time periods (four 15-min sessions or two 30-min sessions vs. one 1-h session) [49]. Since humans spread their daily food intake across multiple meals, studies of circuits regulating feeding behavior per se in the ABA model may not be translationally relevant. On the other hand, the perseverative running could reflect a broader pattern of repetitive behavior, as mice exposed to the ABA paradigm exhibit other perseverative behaviors, such as increased marble burying as well [50], the most reliable assay to measure perseverative behavior in rodents [51–53].

The ABA paradigm has been criticized for its failure to recapitulate aspects of AN such as sex differences, genetic susceptibility, anxiety, social phobias, harm avoidance and fear, and avoidance of dietary fat [6]. It may be that narrowing the scope of what ABA is modeling can be viewed as a strength, because it accelerates direct comparisons with observations in humans. Recapitulation of the link between D2R overexpression and hyperactivity of prefrontal cortical to nucleus accumbens connections and cognitive inflexibility in the ABA model [27••, 39••] opens the door to discover the molecular pathways underlying this key feature of AN [45–47]. If the ABA paradigm does, in fact, primarily capture cognitive inflexibility and perseverative behavior, circuits identified in this model could contribute to both AN and obsessive–compulsive disorder (OCD) [54, 55]. In support of this idea, compulsivity contributes to the variance in eating disorders and OCD and is associated with gray matter volume in the orbitofrontal cortex, ventral striatum, and dorsolateral prefrontal cortex [56]. Neural circuits and molecular pathways uncovered in mouse models could lead to the identification of novel transdiagnostic therapeutic targets.

This review focuses on recent evidence that the ABA paradigm captures both behavioral and neuronal signatures associated with cognitive inflexibility and preservative behaviors observed in AN. This does not exclude the possibility that it can also be used to model metabolic [57–59] or psychological [60–63] adaptations to chronic food restriction and weight loss that promote susceptibility to AN [64–67]. The studies highlighted here provide a roadmap for leveraging the current neuroscience toolbox to demonstrate construct validity in animal models of AN.

## Conclusions

To date, the goal of preclinical studies has been to develop a model that recapitulates as many of the features of AN as possible. While additional control groups in rodents can be used to parse causative factors from secondary consequences of AN, in the end, the sheer complexity of these paradigms makes these analyses extremely expensive and tedious. The power of preclinical research lies in the ability to assess the consequences of targeted manipulations of neuronal circuits that have been implicated in clinical research. Therefore, the primary obstacle to preclinical studies of AN is not the lack of a unitary model that fully recapitulates the disease in humans, but rather the absence of complementary models that capture other features of AN that are not seen in ABA. Better communication between preclinical and clinical researchers is needed to develop translationally relevant paradigms that can be used in cross-species studies to examine how changes in specific brain circuits influence susceptibility to AN.
